# Reply to Stip and colleagues

**DOI:** 10.1016/j.scog.2026.100429

**Published:** 2026-03-19

**Authors:** Torill Ueland, Kristoffer Grimstad, Håkon Sørensen, Merete Glenne Øie, Anja Vaskinn

**Affiliations:** a Section for Clinical Psychosis Research, Division of Mental Health and Addiction, Oslo University Hospital, Oslo, Norway; b Department of Psychology, University of Oslo, Oslo, Norway; c Department of Neurology, Akershus University Hospital, Lørenskog, Norway; d Department of Research, Innlandet Hospital Trust, Brumunddal, Norway; e Center for Research and Education in Forensic Psychiatry, Oslo University Hospital, Oslo, Norway; f Centre for Precision Psychiatry, Division of Mental Health and Addiction, Institute of Clinical Medicine, University of Oslo, Oslo, Norway

We would like to express our gratitude to Stip and colleagues (Stip et al., 2025) for their thoughtful letter commenting on our paper, ‘Subjective cognition in schizophrenia and bipolar disorder: investigation of group differences and associations with objective cognition and clinical characteristics using a novel measure of subjective cognition’ (Grimstad et al., 2025). In their letter they also expand the discussion to include cross-cultural work with the SSTICS, other clinical populations, and the transdiagnostic nature of cognitive complaints. We appreciate their positive feedback and would like to take the opportunity to clarify certain aspects of our work and to discuss some of the other issues raised in the letter.

In our paper (Grimstad et al., 2025), we introduce the Subjective Assessment of Cognitive Complaints Scale (SACCS), a new measure of cognitive complaints. The SACCS includes items aimed to capture the ecological experience of attention, learning and memory, processing speed, and executive functioning in daily life. In their letter Stip and colleagues highlight an important oversight in our study: we did not provide the wording of the questions posed to participants. As they note, this information is crucial for understanding the underlying construct of the SACCS and differentiating it from other validated scales such as the Subjective Scale to Investigate Cognition in Schizophrenia (SSTICS) (Stip et al., 2003) and the Screen for Cognitive Impairment in Psychiatry (SCIP) (Purdon, 2005). We acknowledge their perspective and agree on the importance of transparency. The specific items of the SACCS, along with the theoretically hypothesized constructs they are intended to measure, are presented in Table 1. A detailed, item-by-item comparison of SACCS with SSTICS and SCIP, as suggested by Stip et al. (2025), would indeed be informative for future work. Such cross-scale mapping was beyond the scope of our original paper, but we agree it would be valuable for clarifying overlap and unique contributions of the different instruments.

**Table 1:** SACCS items.

ATTENTION
1. Do you have difficulties concentrating when doing boring tasks?
2. Do you have difficulties concentrating over longer periods of time?
3. Do you have difficulties registering the content when reading newspapers/ magazines/books or when watching TV?
4. Do you have difficulties staying focused on what you are doing if there is a lot going on around you?
5. Do you have difficulties paying attention to several things at the same time?


MEMORY
6. Do you have difficulties remembering appointments?
7. Do you have difficulties keeping information/instructions in mind long enough to complete what you are doing?
8. Do you have difficulties learning new things?


PROCESSING SPEED
9. Do you have difficulties following a conversation?
10. Do you find it hard to grasp the content in a movie/follow subtitles because the pace is too fast?
11. Does it take you a long time to complete tasks at work, school, home?


EXECUTIVE FUNCTIONING
12. Do you have difficulties keeping your finances in order?
13. Do you have difficulties keeping your things in order?
14. Do you have difficulties alternating between different tasks?
15. When you are trying to solve a task, do you find it hard to plan the order in which to do things?
16. Do you find it hard to get started on tasks on your own?
17. Do you often start on tasks without completing them?
18. When you get stuck do you think it's hard to find other ways to solve the task?

In our study we compared objective cognition and subjective cognitive complaints between participants with schizophrenia (SZ), bipolar disorder (BD) and healthy controls (HC). In accordance with previous research (Bortolato et al., 2015) the SZ group performed worse than HCs and the BD group performed at an intermediate level on objective cognitive measures (MCCB). On the subjective complaints measure, the SACCS, participants with SZ and BD both reported more complaints than HCs but with no difference between the clinical groups. This is line with Stip et al.'s (2025) findings. However, it was the BD group (SACCS total: 34.3) that had numerically more subjective cognitive complaints than the SZ group (SACCS total 33.6), not the other way around as was the case in Stip et al.'s (2025) study. Overall, the picture is, however the same: level of subjective cognitive complaints does not appear to differentiate between schizophrenia and affective disorders.

In their letter, Stip and colleagues further stress the need for more research into the relationship between cognitive complaints, mood and other clinical variables and suggest that subjective complaints may have different associations to insight in these two clinical groups. Our study provided some information that this may indeed be the case. It did not survive correction for multiple comparisons, but an initial association between poorer insight and fewer cognitive complaints was seen in the SZ, but not in the BD group (Grimstad et al., 2025). For the BD group, cognitive complaints were linked to a global measure of psychopathology and functioning.

Finally, Stip et al. (2025) discuss introspective accuracy (IA), i.e. self-assessment of cognitive abilities. IA can be measured by asking participants to rate their performance on objective cognitive tests and has been found to be impaired in BD and SCH (Morgan et al., 2022). Our measure, the SACCS does not require participants to rate their performance on cognitive tests but rather measures their subjective experience of cognitive functioning in daily life. Although not strictly an IA score, we have now computed discrepancy scores between objective (MCCB) and subjective (SACCS) cognitive test scores. We used the means and standard deviations of our healthy control group to calculate z-scores for the MCCB and SACCS total scores, noting that higher SACCS scores indicate more complaints (positive z-scores) and lower MCCB performance indicates greater impairment (negative z-scores). Finally, we computed discrepancy scores by subtracting the MCCB z-score from the SACCS z-score. See Table 2.

**Table 2:** Objective cognition, subjective complaints, discrepancy scores, education, and IQ.

	SZ	BD
MCCB composite, M (SD)	−0.84 (0.61)	−0.40 (0.54)
SACCS composite, M (SD)	1.97 (1.33)	2.05 (1.44)
Discrepancy MCCB-SACCS	1.13	1.65
Educational attainment, M (range)	12.37 (9–18)	13.55 (9–17)
IQ, M (SD)	101.49 (12.49)	111.12 (11.58)

Abbreviations: MCCB: Matrics Consensus Cognitive Battery, SACCS: Self Assessed Cognitive Complaints,

We find that the BD participants exhibit a larger discrepancy than SZ participants, a difference that approached statistical significance (*t* = −1.883, *p* = 0.063). A possible explanation is that the BD group's higher educational attainment and IQ (see Table 2), indicative of better premorbid functioning, has led them to perceive a larger decline after illness onset which is reflected in a greater discrcrepancy. This interpretation is consistent with the observed associations between BD participants' subjective cognitive scores and relationship with global psychopathology and functioning. This hypothesis can however not be adequately tested with the present data and would require longitudinal or premorbid measures in future studies. A substantial difference between objectively and subjectively assessed cognition, whether operationalized as discrepancy scores or as introspective accuracy, indicates the potential of psychological interventions to overcome defeatist attitudes towards one's own cognitive potential.

Finally, we strongly agree with Stip and colleagues' view that measuring cognitive functioning both objective and subjective is very important. Unfortunately, although cognitive assessment is recommended in many national and international guidelines for both SZ and BD, many people with these illnesses still do not receive an assessment (Bryce et al., 2024).

## CRediT authorship contribution statement

**Torill Ueland:** Conceptualization, Formal analysis, Writing – original draft, Methodology. **Kristoffer Grimstad:** Data curation, Writing – review & editing. **Håkon Sørensen:** Data curation, Writing – review & editing. **Merete Glenne Øie:** Writing – review & editing. **Anja Vaskinn:** Conceptualization, Writing – review & editing.

## Funding information

This study was funded by the 10.13039/501100005416Research Council of Norway (grant number 223273). The funding source had no involvement in the study design or preparation of the article.

## Declaration of competing interest

The authors declare that they have no known competing financial interests or personal relationships that could have appeared to influence the work reported in this paper.

## References

[bb0005] Bortolato B., Miskowiak K.W., Köhler C.A., Vieta E., Carvalho A.F. (2015). Cognitive dysfunction in bipolar disorder and schizophrenia: a systematic review of meta-analyses. Neuropsychiatr. Dis. Treat..

[bb0010] Bryce S., Cheng N., Dalton A., Ojinnaka A., Stainton A., Zbukvic I., Ratheesh A., O’Halloran C., Uren J., Gates J., Daglas-Georgiou R., Wood S.J., Allott K. (2024). Cognitive health treatment priorities and preferences among young people with mental illness: the your mind, your choice survey. Early Interv. Psychiatry.

[bb0015] Grimstad K., Sørensen H., Vaskinn A., Mohn C., Olsen S.H., Andreassen O.A., Lagerberg T.V., Melle I., Øie M.G., Ueland T., Haatveit B. (2025). Subjective cognition in schizophrenia and bipolar disorder: investigation of group differences and associations with objective cognition and clinical characteristics using a novel measure of subjective cognition. Schizophr. Res. Cogn..

[bb0020] Morgan O., Strassnig M.T., Moore R.C., Depp C.A., Ackerman R.A., Pinkham A.E., Harvey P.D. (2022). Accuracy of immediate self-assessment of neurocognitive test performance: associations with psychiatric diagnosis and longitudinal psychotic symptoms. J. Psychiatr. Res..

[bb0025] Purdon S.E. (2005).

[bb0030] Stip E., Caron J., Renaud S., Pampoulova T., Lecomte Y. (2003). Exploring cognitive complaints in schizophrenia: the subjective scale to investigate cognition in schizophrenia. Compr. Psychiatry.

[bb0035] Stip E., Al Mugaddam F., Abdel Aziz K., Javaid S.F., Nauman J., ElBarazi I., Potvin S., Tourjman V., White N. (2025). Subjective cognition in schizophrenia and bipolar disorder: complaints with SSTICS & SACCS. Schizophr. Res. Cogn..

